# Plasma Vitamin D Status and Its Correlation with Risk Factors of Thrombosis, P-selectin and hs-CRP Level in Patients with Venous Thromboembolism; the First Study of Iranian Population 

**Published:** 2014

**Authors:** Taher Entezari-Maleki, Azita Hajhossein Talasaz, Mojtaba Salarifar, Molouk Hadjibabaie, Mohammad Reza Javadi, Ali Bozorgi, Yaser Jenab, Mohammad Ali Boroumand, Kheirollah Gholami

**Affiliations:** a *Department of Clinical Pharmacy, Faculty of Pharmacy, Tehran University of Medical Sciences, Tehran, Iran. *; b *Department of Clinical Pharmacy, Faculty of Pharmacy, Tabriz University of Medical Sciences, Tabriz, Iran. *; c *Department of Cardiology, Tehran Heart Center, Tehran University of Medical Sciences, Tehran, Iran. *; d *Department of Clinical pharmacy, faculty of Pharmacy and Research Center for Rational Use of Drugs, Tehran University of Medical Sciences, Tehran, Iran. *; e *Department of Molecular Pathology, Tehran Heart Center, Tehran University of Medical Sciences, Tehran, Iran. *

**Keywords:** Vitamin D deficiency, DVT, PE, VTE, P-selectin, Hs-CRP, Thrombosis risk factors

## Abstract

Low plasma level of vitamin D is linked to the increased risk of cardiovascular diseases such as hypertension, diabetes, dyslipidemia and peripheral vascular diseases. Vitamin D deficiency is a worldwide problem that involves Iranian population. To the best of our knowledge, this was the first investigation on venous thromboembolism (VTE) subjects that assessed the correlation of vitamin D level with plasma P-selectin, hs-CRP, and risk factors of thrombosis.

In this prospective pilot study, patients with diagnosis of acute deep vein thrombosis and/ or pulmonary embolism were enrolled. All patients’ clinical data, demographics and risk factors of thrombosis were evaluated. Plasma level of P-selectin and hs-CRP were measured by ELISA method. Radio immune assay method was used to determine plasma level of 25-hydroxy vitamin D (25(OH) D). In this study, 60 subjects were included. The mean ± SD plasma 25-hydroxy vitamin D level (25(OH) D) of participants was 21.4 ± 14.6 ng/mL. The vitamin D deficiency was detected in 60% of patients. No significant relation was found between the plasma 25(OH)D level and P-selectin and hs-CRP. In multiple regression analysis, there was a significant relationship between the level of 25(OH)D and the patients’ age (beta = 0.452; p = 0.001), diabetes (beta = 0.280; p = 0.036) and positive family history of cardiovascular diseases (beta = 0.373; p = 0.003).

Vitamin D deficiency is a frequent problem in Iranian VTE patients. Moreover, Plasma level of vitamin D is not associated with P-selectin and hs-CRP in VTE patients.

## Introduction

Vitamin D deficiency, defined as a plasma 25-hydroxyvitamin D3 (25(OH) D) level under 20 ng/mL, is highly prevalent with an incidence of about 30-50% in all over of the world (1-3). A low level of vitamin D is linked to the increased risk of cardiovascular diseases (CVD) and mortality (2, 4-6).

Currently, the potential effect of vitamin D on the cardiovascular system has been elucidated. Moreover, the association of vitamin D deficiency and the incidence of thromboembolism is described in various studies (7-10).

The main suggested mechanisms for anti-thrombotic properties of vitamin D including “up-regulation of thrombomodulin” (9, 11, 12) and “down-regulation of tissue factor” (TF) (9, 12).

In addition, vitamin D can up-regulate and increase the level of anti-inflammatory cytokine of IL10 (13, 14). Furthermore, inflammation is also a known cause of coagulation with high sensitivity C-reactive protein (hs-CRP) as the main involved inflammatory cytokine (15, 16). 

P-selectin, a family part of the lectin protein with a procoagulant characteristic is stored in the alpha granules of platelets and Weibel-Palade bodies of endothelial cells (17, 18). After platelet activation, soluble form of P-selectin (sP-selectin) distributes to the blood and triggers the coagulation pathway by binding to its receptor P-selectin specific ligand-1 (PSGL-1) placed on the outside of leukocytes and platelets (17, 18). According to literature reports, plasma level of sP-selectin is highly associated with the incidence of venous thromboembolism (VTE) (18-23).

To the best of our knowledge, the association between vitamin D and P-selectin and hs-CRP has not yet been reported in studies. Therefore, this study was performed to investigate correlation of vitamin D level with plasma P-selectin, hs-CRP and risk factors of thrombosis in these subjects. 

## Experimental

*Study design*

In a prospective study, in Tehran Heart Center (THC) all consecutive patients with the diagnosis of acute deep vein thrombosis (DVT) and/or pulmonary embolism (PE) were enrolled to the study from June to September of 2012. Ethic committee of the university approved this study. The recruitment criteria for this study were all consented patients with 18 years old and over, with diagnosis of DVT and/or PE that were confirmed by color duplex sonography and Computed Tomography pulmonary angiography (CTPA) respectively.

A data collecting form was designed by the investigators in order to record patients’ information, which included patients’ demographic data, such as sex, age, weight, height, body mass index (BMI), past medical history, drug history, laboratory data, and Positive family history of CVD defined as “a premature coronary heart disease including definite myocardial infarction or sudden death (before 55 years for men and before 65 years for women) in first-degree relatives” (24). Additionally, risk factors of thrombosis including history of cancer, surgery, obesity (is defined as a BMI more than 30 Kg/m2), estrogens use, smoking, hypertension, diabetes, advanced age, and hyperlipidemia were recorded (25). The exclusion criteria included coexistence of autoimmune and inflammatory diseases, pregnancy, any positive history of immunosuppressant, immunomodulator medications consumption, hypercalcemia, renal failure, sarcoidosis, lymphoma, abnormalities of 1-alphahydroxylase, hypophosphatic rickets or vitamin D receptor defects.

*Blood sampling*

At the time of acute DVT or PE, blood samples were achieved from an antecubital vein of participants, and then were drawn into 3.2% sodium citrated blood collection tubes. Blood specimens were spun at 1500 × rpm for 10 minutes, at room temperature. The plasma was drawn off and aliquotted into 1- mL microtubes, and then frozen and stored at −25 °C for measurements of the 25(OH) D, P-selectin and hs-CRP levels. 

*Plasma 25-hydroxyvitamin D, Soluble P-selectin and high sensitive C - reactive protein (hs-CRP) measurement*

Enzyme-linked immunosorbent assay (ELISA) test was carried out for quantitative detection of human plasma sP-selectin level (Wuhan Boster Biological Technology Co. Ltd, Wuhan City, China*) *and hs*-*CRP (Pars Azmun Co., Karaj, Iran). Plasma level of 25(OH) D was determined by using DiaSorin radioimmunoassay (RIA) method (DiaSorin Corporation, Stillwater, MN). The quantities were reported in ng/mL for sP-selectin and 25(OH) D and mg/L for hs-CRP. 

The 25(OH) D levels under 20 ng/mL were regarded as vitamin D deficiency; concentrations between ≥20-30 ng/mL were deemed as vitamin D insufficiency; and 30 ng/ mL and more were considered as vitamin D sufficiency (1, 5). For assessment of correlation of vitamin D deficiency with P-selectin, hs-CRP and thrombosis risk factors, all patients were allocated in two groups of vitamin D deficient (25(OH)D <20 ng/mL) and non-deficient (25(OH)D 20 ng/mL). The plasma hs-CRP levels of more than 10 mg/L were considered very high (16). The P-selectin levels of less than 60 ng/mL were considered to rule out the VTE and levels of more than 90 ng/mL were deemed to confirm VTE (22). 

*Statistical analysis*

Data analysis was conducted in SPSS 16. Kolmogorov–Smirnov test was performed to determine if data had a normal distribution. Spearman test was used for correlation evaluation between continuous variables. Mann–Whitney (for non-parametric variables) and independent-sample t-test (for parametric variabels) was used for the assessment of any potential link between the level of vitamin D and P-selectin, hs-CRP levels, patients’ demographic data, or laboratory findings. In addition, one-sample t-test and Wilcoxon signed ranks test were used to compare 25(OH) D, p-selectin, and hs-CRP levels with their normal range values. Chi-square and Fisher exact test was applied for frequency analysis. Odds ratios were calculated for risk estimation between vitamin D deficient and non-deficient groups. Linear Regression was also performed to assess the correlation between the risk factors of thromboembolism and study variables with vitamin D level. p-values less than 0.05 was regarded as significant. 

## Results

In this study, 60 subjects including 32 (53.3%) males and 28 (46.7%) females with the diagnosis of acute DVT (n = 35) and PE (n = 25) were enrolled. The mean ± SD of patients’ age was 54.7 ± 16.5 years old. The mean plasma 25(OH) D level of participants was 21.4 ± 14.6 ng/mL. The result of this investigation revealed vitamin D deficiency in 60% (n=36) of patients. Additionally, 23.3% (n = 14) of them were vitamin D insufficient, and only 16.7% (n = 10) had normal levels of vitamin D. The mean plasma P-selectin and hs-CRP level of patients were 120.5 ± 34.4 ng/mL and 31.6 ± 45.4 mg/L, respectively. There was no significant difference between 25(OH)D levels of obese and non obese patients. 

As mentioned, in patients with thromboembolism, the mean plasma level of 25(OH) D was significantly lower than normal cutoff of 30 ng/mL (p=0.001; 95% CI:-12.39- -4.86). In this line, the mean plasma level of P-selectin was higher than cutoff of 90 ng/mL (p = 0.001; 95% CI: 21.6 – 39.36). As well, the mean plasma hs-CRP level was significantly higher than very high cut-point of 10 mg/L (Wilcoxon signed ranks test; p = 0.023). However, 46.6% (28 participants) had hs-CRP levels of below 10 mg/L. 

We could not find any significant correlation between plasma level of P-selectin and hs-CRP with 25 (OH) D levels. Moreover, no significant differences in P-selectin, hs-CRP, and 25(OH) D levels between males and females and DVT and PE subjects were found. 

Demographics and clinical data of patients in two groups of vitamin D deficient and non-deficient are shown in Table 1. 

**Table 1:** Demographic and clinical data of vitamin D deficient and non-deficient patients (n = 60).

**Demographic / Clinical date**	**Vitamin D deficient group ** **(n = 36)**	**Vitamin D non-deficient group (n = 24)**	**p-value**
Age (years), mean ± SD	49.2 ± 14.8	63 ± 15.5	0.001
Sex, male (%), mean ± SD	22 (61.1)	10 (41.6)	0.189
Weight (Kg), mean ± SD	76.1 ± 15.8	72.7 ± 11.7	0.514
Height (cm), mean ± SD	169.4 ± 13.1	161.4 ± 9.5	0.070
BMI (Kg/m2), mean ± SD	27.1 ± 5.7	27.9 ± 4.3	0.667
25-hydroxy vitamin D (ng/mL) , mean ± SD	12.7 ± 3.5	34.3 ± 15.3	0.001
P-selectin(ng/dL), mean ± SD	125 ± 35.9	113.7 ± 31.1	0.215
High sensitivity C-reactive protein (mg/L), mean ± SD	36.7 ± 52.5	24 ± 31.3	0.629
Serum creatinine (mg/dL), mean ± SD	0.93 ± 0.3	1.31 ± 1	0.057
Blood urea nitrogen (mg/dL), mean ± SD	33 ± 16.5	48.2 ± 31.9	0.026
Fasting blood sugar (mg/dL), mean ± SD	117.3 ± 59.8	112.5 ± 34.5	0.577
Triglyceride (mg/dL), mean ± SD	147.2 ± 61.3	142.1 ± 53.3	0.769
Total cholesterol (mg/dL), mean ± SD	154.1 ± 51.5	165 ± 49	0.480
High density lipoprotein (mg/dL), mean ± SD	33.7 ± 9.5	37.4 ± 14.2	0.291
Low density lipoprotein (mg/dL), mean ± SD	107.6 ± 40.4	103 ± 34	0.684
Very low density lipoprotein (mg/dl), mean ± SD	30.9 ± 12.5	28.7 ± 10.8	0.551
White blood cell (/mm3), mean ± SD	10738 ± 4596.2	9237.2 ± 2665.7	0.215
Red Blood cell (/mm3), mean ± SD	4.91 ± 0.79×105	4.87 ± 1.48×105	0.919
Hemoglobin (g/dL), mean ± SD	13.3 ± 3.2	12.7 ± 2.6	0.497
Platelet(/mm3), mean ± SD	268970 ± 14097	229500 ± 83863.5	0.289
Diabetes, n (%)	4 (11.1)	6 (25)	0.187
Ischemic heart disease, n (%)	2 (5.5)	7 (29.1)	0.009
Hypertension, n (%)	6 (16.6)	9 (37.5)	0.126
Hyperlipidemia, n (%)	7 (19.4)	6 (25)	0.751
Orthopedic surgery, n (%)	3 (8.3)	0 (0)	0.268
Malignancy, n (%)	2 (5.5)	0 (0)	0.512
Positive family history, n (%)	3 (8.3)	6 (25)	0.187
Drug history of oral contraceptive pills, n (%)	4 (11.1)	1(4.1)	0.639
Drug history of cardiovascular agents, n (%)	12(33.3)	16 (66.6)	0.017
Drug history of antiglycemic agents, n (%)	2(5.5)	4 (16.6)	0.028
Deep vein thrombosis, n (%)	21(58.3)	14 (58.3)	1.000
Pulmonary embolism, n (%)	15 (41.7)	10 (41.7)	1.000
History of smoking, n (%)	6 (16.6)	1 (4.1)	0.223

In correlation evaluation with Spearman test, 25(OH) D levels significantly correlated with age (r = 0.516; P = 0.001), P-selectin levels significantly correlated with age (r = -0.277; P = 0.032). Hs-CRP significantly correlated with high-density lipoprotein (HDL) (r = -0.334; p =0.020). Although there was a negative correlation between 25 (OH) D levels and P-selectin (r = -0.186; p = 0.155) or hs-CRP (r = - 0.063; p = 0.630), these correlations were not significant. 

Odds ratio for risk factors of thromboembolism were calculated in vitamin D deficient and non-deficient groups. The results were shown in Table 2. 

**Table2:** Odds ratio of thrombosis risk factors in vitamin D deficient and non-deficient patients

	**Odds ratio**	**95% Confidence Intervals**	**p-value**
Diabetes Mellitus	0.44	0.11- 1.74	0.244
Ischemic Heart Disease	0.19	0.03 - 0.99	0.049
Hypertension	0.44	0.140 - 1.41	0.168
Hyperlipidemia	0.77	0.23 - 2.59	0.683
Malignancy	3.35	0.15 - 72.97	0.440
Orthopedic surgery	4.69	0.23 - 95.05	0.313
Positive family history	1.4	0.31- 6.23	0.658
Oral contraceptive pill	2.87	0.3 – 27.43	0.358
Smoking history	4.00	0.45 - 35.35	0.212

In multiple linear regression analysis between thrombosis risk factors and vitamin D levels, statistically significant relationship were found between patients’ age, positive family history of cardiovascular disorders and diabetes mellitus with vitamin D levels as shown in Table 3. Furthermore, in the regression analysis between all study variables and vitamin D levels, hypertension was the predictor factor and significantly linked with vitamin D levels (beta = 0.761; p = 0.028, 95 % CI: 2.78 - 34.52).

**Table 3:** Results of multiple regression analysis between thrombosis risk factors and vitamin D levels

**Model ** **no.**	**Model ** **Predictor(s)**	**Factors**	**Beta**	**SE**	**P**	**R** **2**	**Adjusted R** **2**	**95% Confidence Interval for B**
1	Age	Age Diabetes mellitus Family history P-selectin hs-CRP	0.452 0.280 0.373 0.053 -0.166	0.120	0.001 0.036 0.003 0.705 0.199	0.205	0.188	0.180-0.661
2	Age and Family History	Age Family history P-selectin hs-CRP	0.408 0.373 -0.049 -0.193	0.111 4.942	0.001 0.003 0.714 0.104	0.342	0.314	0.157-0.603 5.510-25.392

## Discussion

To the best of our knowledge, this is the first report from Iran that has been investigated vitamin D status and its correlation with thrombosis risk factors, P-selectin and hs-CRP in VTE subjects.

As mentioned before, low levels of vitamin D is associated with the increased risk of cardiovascular diseases and mortality (2, 4-6). In this study, the majority of patients were vitamin D deficient. This finding is compatible with the various large-scale studies described vitamin D deficiency as a risk factor of thrombosis (7, 10). However, in healthy population of Tehran, the place of doing this study, the prevalence of severe, moderate, and mild Vitamin D deficiency was reported as “9.5%, 57.6%, and 14.2%” respectively (26). In addition, the prevalence of vitamin D deficiency in the same geographical zone, in healthy population of Saudi Arabia, the United Arab Emirates, Turkey, and Lebanon was reported as 30-50% (1). Therefore, the prevalence of vitamin D deficiency in the healthy subjects is also very high. 

Based on these results, well designed, large-scale studies were needed to clearly determine the main role of vitamin D deficiency as a risk factor of thrombosis in patients with thromboembolism. 

We used level of 25-hydroxy vitamin D to measurement of vitamin D status. Because of this circulating type of vitamin D is the best indicator of vitamin D supply in body. Moreover, 25(OH) D level was used for detection of vitamin D deficiency. However, based on Ray *et al*. report in some conditions including “vitamin D receptor defects, abnormalities of 1-alphahydroxylase, hypercalcemia, hypophosphatic rickets, renal failure, sarcoidosis and lymphoma” w 1,25-dihydroxy vitamin D testing is the preferred method to measurement of vitamin D levels in the body (27). Furthermore, these conditions were our exclusion criteria.

P-selectin is an adhesion cell glycoprotein with a procoagulant activity that increases in the venues thromboembolism conditions. In accordance with available data, P-selectin is procoagulant factor and a known biomarker of thrombosis with high level of sensitivity and specificity for diagnosis of VTE (18-20, 22, 23, 28). Ramacciotti *et al*. described a combination of P-selectin levels with cut-point ≥ 90 ng/mL and Wells criteria score ≥ 2 as the diagnostic criteria of DVT with a specificity of 96% and positive predictive value of 100%. Furthermore, a cut-point of under 60 ng/mL of P-selectin and Wells scores of under 2 could rule out the diagnosis of DVT with 99%, 33% and 96% of sensitivity, specificity and negative predictive value, respectively (22). In our study, the mean P-selectin levels in DVT and PE patients were significantly higher than cut-point of 90 ng/mL. This finding is in line with other studies, which were reported the P-selectin as a predictive factor of VTE and its increased levels in thrombosis condition (18-20,22,23,28). Additionally, level of P-selectin in DVT and PE subjects was not statistically different. This finding is compatible with Ay *et al*. report (20).

hs-CRP is one of the main inflammatory cytokine participated in development of thrombosis (15, 16). The mean hs-CRP level of participants was significantly higher than cut-point of 10 mg/L; this result may demonstrate the existence of inflammation as a promoting factor of thrombosis in patients with venous thromboembolism. Recently, data also showed that anticoagulant therapy can decrease significantly the level of hs-CRP in atrial fibrillation patients (29). 

We could not find any significant relationship between vitamin D and P-selectin and hs-CRP levels; however, this correlation was negative. There is still a lack of data about relation between P-selectin as a procoagulant factor and vitamin D with antithrombotic properties. As mentioned above, P-selectin increases in thrombosis status and based on Ay *et al*. study independently can predict thrombosis in cancer patients with VTE (19). Furthermore, large-scale studies are needed to obviously describe the association between vitamin D and P-selectin and hs-CRP in VTE patients. 

The inverse significant association between HDL and hs-CRP is another finding of this study, which is consistent with other reports (30, 31). The cardioprotective effect of HDL has been described and low HDL levels are demonstrated as one of the main risk factor of coronary heart diseases (CHD) (32). Additionally, HDL has antithrombotic properties and can prevent arterial and venous thrombosis (33). Therefore, low levels of HDL and coexistence of inflammation can result in thrombosis formation.

The results of this study showed an inverse significant relationship between P-selectin levels and patients’ age that is in agreement with Barbaux report in patients with coronary artery disease (CAD) (34). In this report, P-selectin levels in patients older than 65 years were significantly lower than healthy individuals. The authors proposed two interpretations for this finding. First, high level of P-selectin in elderly patients with CAD is associated with increased mortality rate. Therefore, this inverse relation could be elucidating in survivors. The second explanation is that P-selectin may play various effects along with the development of atherosclerosis. It has been shown that sP-selectin can bind to leukocytes via PSGL-1 or sialyl Lewis X without activating their consequent enrollment on the vascular surface. Therefore, this can limit the over activation of leukocytes (34-36).

Multiple regression analysis showed a significant relation between age, positive family history of cardiovascular disease and diabetes mellitus with vitamin D levels. Moreover, hypertension was a predictor factor and significantly correlated with vitamin D levels. The inverse relation of 25(OH)D levels with hypertension and diabetes mellitus was described in large-scale study of NHANES III national cohort registry (37) and other cross-sectional studies (5, 38, 39). Vitamin D deficiency causes to “up-regulation of renin-angiotensin-aldosterone system” and results in hypertrophy of cardiovascular smooth muscle cells (5, 40). Furthermore, the active metabolite of vitamin D suppresses renin production, that can lower blood pressure (5, 41). Direct inhibition of nuclear factor-jB (NF-jB) pathway (42) and “secondary hyperparathyroidism prevention” are other anti-hypertensive mechanisms of vitamin D (43). 

Based on Chiu *et al*. investigation, vitamin D deficiency leads to “insulin resistance and beta cell dysfunction” (44). Moreover, Vitamin D supplementation reduces the risk of both diabetes 1 and 2 (45, 46). Thus, in diabetic patients low levels of vitamin D can deteriorate the disease condition and results in more complications such as promoting of thrombosis. 

A significant A significant link between the family history of cardiovascular disorders and vitamin D levels was another finding of this study that is in line with other reports (47). The role of genetic variation on vitamin D status in individuals may elucidate this result. vitamin D transportation was regulated by three loci that are also corresponded to cholesterol synthesis and hydroxylation (48). Furthermore, in a Caucasian subpopulation, genetic variation on“rs2762933in CYP24A1 and“rs6055987 in PLCB1” were recognized as an independent indicator of vitamin D status (49).

According to MacLaughlin and Holick report, the skin synthesis of vitamin D is reduced with aging and this decline significantly occurred from the individuals 77- and 82 years old (50). In multiple linear regression analysis, we also reported the positive significant relation between age and vitamin D level. However, in this study, lower age of participants and sample size may partially justify this result.

The small sampling size, time, and cost constraint can be our limitations in this study.

In conclusion, the majority of subjects had a low level of 25(OH) D at the time of acute DVT or PE. Diabetes mellitus, hypertension, age, and positive family history of cardiovascular disorders significantly correlated with low vitamin D levels in this population. Moreover, Plasma level of vitamin D is not associated with P-selectin and hs-CRP in VTE patients. Further studies are needed to confirm this finding.
